# Mutants of *Yarrowia lipolytica* NCIM 3589 grown on waste cooking oil as a biofactory for biodiesel production

**DOI:** 10.1186/s12934-017-0790-x

**Published:** 2017-10-24

**Authors:** Gouri Katre, Namasvi Ajmera, Smita Zinjarde, Ameeta RaviKumar

**Affiliations:** 1 Institute of Bioinformatics and Biotechnology, Savitribai Phule Pune University, Pune, 411 007 India; 2 Department of Biotechnology, Savitribai Phule Pune University, Pune, 411 007 India

**Keywords:** *Yarrowia lipolytica*, Chemical mutagenesis, Cerulenin, Lipid production, Biodiesel, Waste cooking oil

## Abstract

**Background:**

Oleaginous yeasts are fast emerging as a possible feedstock for biodiesel production. *Yarrowia lipolytica*, a model oleaginous yeast is known to utilize a variety of hydrophobic substrates for lipid accumulation including waste cooking oil (WCO). Approaches to increase lipid content in this yeast include metabolic engineering which requires manipulation of multiple genes in the lipid biosynthesis pathway. A classical and cost-effective approach, namely, random chemical mutagenesis on the yeast can lead to increased production of biodiesel as is explored here.

**Results:**

In this study, chemical mutagenesis using the alkylating agent, *N*- methyl-*N′*-nitro-*N*-nitrosoguanidine (MNNG) as well as an additional treatment with cerulenin, a fatty acid synthase inhibitor generated 800 mutants of *Y. lipolytica* NCIM 3589 (761 MNNG treated and 39 MNNG + cerulenin treated). A three-stage screening using Sudan Black B plate technique, Nile red fluorimetry and total lipid extraction using solvent was performed, which enabled selection of ten high lipid yielding mutants. Time course studies of all the ten mutants were further undertaken in terms of biomass, lipid yield and lipid content to select three stable mutants (YlB6, YlC7 and YlE1) capable of growing and accumulating lipid on WCO, with lipid contents of 55, 60 and 67% as compared to 45% for the wild type. The mutants demonstrated increased volumetric lipid productivities (0.062, 0.044 and 0.041 g L^−1^ h^−1^) as compared to the wild type (0.033 g L^−1^ h^−1^). The fatty acid profile of the three mutants consisted of a high content of C16 and C18 saturated and monounsaturated fatty acids and was found to be suitable for biodiesel production. The fuel properties, namely, density, kinematic viscosity, total acid number, iodine value of the three mutants were evaluated and found to lie within the limits specified by internationally accepted standards. Additionally, it was noted that the mutants demonstrated better cetane numbers and higher heating values than the wild type strain.

**Conclusion:**

The chemical mutagenesis strategy adopted in this study resulted in the successful isolation of three stable high SCO yielding mutants. The mutants, namely, YlB6, YlC7 and YlE1 exhibited a 1.22, 1.33 and 1.49-fold increase in lipid contents when grown on 100 g L^−1^ waste cooking oil than the parental yeast strain. The fatty acid methyl ester (FAME) profiles of all the three mutants was determined to be suitable for biodiesel suggesting their potential applicability while simultaneously addressing the management of waste cooking oil.

**Electronic supplementary material:**

The online version of this article (doi:10.1186/s12934-017-0790-x) contains supplementary material, which is available to authorized users.

## Background

Biodiesel, an alternative energy source, consists of mono-alkyl esters of long-chain fatty acids (fatty acid methyl esters, FAMEs) produced by transesterification of plant, microbial and algal oils or animal fats. The advantages of biodiesel include its renewable nature, ease of manufacture, favourable carbon footprint, biodegradability, good flash point, low sulfur content and compatibility for blending with petrodiesel [1]. The global biodiesel production has increased by 7.5% in 2015 from 28.7 billion liters to 30.8 billion liters in 2016 [2]. Hence, it is necessary to investigate the potential of non-edible oil sources and waste feedstock for expanding biodiesel production in a sustainable manner.

The main economic challenge for successful production of biodiesel is the high cost of the feedstock. Utilization of industrial and domestic waste as substrates by oleaginous fungi for biodiesel production will not only address management of waste but is also a sustainable and potentially low cost approach to improve process economics. One such waste, waste cooking oil (WCO) is generated at 29 million tons per year [3], which is disposed of by direct discharge on land or into water bodies causing environmental harm [4]. WCO which contains high levels of free fatty acids (FFAs) has been used to produce biodiesel by acid, alkali, or enzyme catalysis and via non-catalyst transesterification. Apart from a high content of FFAs and water, WCO also contains glycerides, dimeric and polymeric acids formed during frying [5]. All these components affect the conventional alkali catalyzed transesterification reaction leading to soap formation resulting in lower yields and non-desirable FAME types, which in turn affect the biodiesel properties [6]. The acid catalyzed process is not sensitive to FFA and water content, but the production process requires long time periods of 18–24 h and has an additional disadvantage of salt interactions leading to corrosion. Enzymes used to catalyze transesterification are slower, expensive for large scale production and result in enzyme inactivation in presence of methanol [7]. Also, these heterogeneous acid and enzyme catalyzed systems suffer from mass transfer limitations and are not favorable for large scale applications. Non-catalyst and supercritical methods require high temperature and pressure and are not economical [8].

Due to the above mentioned limitations, which would require pre-treatments, microbial conversion of WCO to SCOs, with a fatty-acid utilizing yeast like *Yarrowia lipolytica* would be the preferred option. Our earlier study [9] shows that these SCOs from *Y. lipolytica* NCIM 3589 can be used as a feedstock for biodiesel. The yeast exhibited a maximal lipid/biomass yield coefficient (0.43 g g^−1^) when grown on WCO in 72 h. Thus, while conversion of yeast biomass into SCO for biodiesel production is a cost effective and eco-friendly process, the constraint is the mediocre lipid content reported for *Y. lipolytica* [4, 10].

Various approaches have been used to increase lipid titers for yeast cultures including genetic engineering and media optimization [11, 12]. Microbial strain improvement, which can be achieved through classical mutagenesis is another effective way to increase SCO production [13] and several mutagens such as Ultraviolet light (UV), ethyl methanesulfonate (EMS) and *N*-methyl-*N′*-nitro-*N*-nitrosoguanidine (MNNG) have been used [14–16]. A co-selection pressure of cerulenin, a fatty acid synthesis inhibitor, along with the chemical mutagen can also be applied to select mutants. Those mutants possessing high fatty acid synthase activity would overcome this inhibition and grow in the presence of cerulenin.

The objective of the present study was to increase the lipid content of *Y. lipolytica* NCIM 3589 by strain improvement using chemical mutagenesis. Mutants were obtained by non-specific mutagenesis using MNNG alone (MNNG) and with an additional selective pressure of cerulenin (MNNG + cerulenin). To make the process more cost effective, the mutants were checked for their lipid accumulation potential on a waste substrate, WCO. As strain improvement by random mutagenesis requires a good screening methodology for selection of high SCO yielding mutants, preliminarily screening was carried out by using Sudan Black B (a lipid stain) and subsequently quantified using Nile red spectrofluorimetry. The mutants showing higher total lipid yield as compared to the wild type were selected and time course studies were carried out to evaluate their biomass, total lipid yield and lipid content. The fatty acid profile and fuel properties of the mutants showing highest lipid content were also determined to check their applicability as a fuel. Molecular typing of the selected mutants was carried out and stability studies were done to ascertain their long term suitability for biodiesel production.

## Methods

### Chemicals

The chemical mutagen, MNNG was purchased from TCI Chemicals, Tokyo, Japan. Cerulenin and Nile red were purchased from Sigma-Aldrich, Inc., USA. Sudan Black B was obtained from Hi-media laboratories, Mumbai, India. All other chemicals used were of analytical grade and > 99% pure as per the manufacturer’s specifications. The waste cooking oil was obtained in bulk from a local eatery in Pune, Maharashtra, India. The oil contained 40.5% palmitic acid, 40.39% oleic acid and 10.35% linoleic acid as determined by GC-FID as per the AOCS method [17].

### Strain and growth conditions

*Yarrowia lipolytica* NCIM 3589 was selected for mutagenesis based on our previous studies [9]. The strain was maintained on MGYP medium (g L^−1^): 3.0 malt extract; 3.0 yeast extract; 5.0 peptone and 10.0 dextrose, at 4 °C. The liquid broth of similar composition was used for development of the pre-inoculum. To evaluate the mutants for their total lipid yield after MNNG mutagenesis, lipid accumulation medium (LAM) was used (g L^−1^): 30.0 glucose, 1.5 yeast extract, 0.5 NH_4_Cl, 5.0 Na_2_HPO_4_·12H_2_O, 7.0 KH_2_PO_4_, 1.5 MgSO_4_·7H_2_O, 0.1 CaCl_2_·2H_2_O, 0.01 ZnSO_4_·7H_2_O, 0.08 FeCl_3_·6H_2_O and (mg L^−1^) 0.1 CuSO_4_·5H_2_O, 0.1 Co [NO_3_]_2_·6H_2_O, 0.1 MnSO_4_·5H_2_O and pH adjusted to 5.5. All shake flask experiments were carried out in 250 mL Erlenmeyer flasks with 100 mL of medium and incubated on a rotary incubator shaker at 120 rpm at 30 °C.

### Effect of cerulenin on colony size

The effect of the fatty acid synthase inhibitor, cerulenin on the colony size of wild type was also determined. A cell count of 2 × 10^8^ cells/mL was taken. The cerulenin stock solution (1 mg/mL) was prepared in absolute ethanol. Different concentrations of cerulenin (0–20 µg/mL) were incorporated into lipid accumulation medium (LAM). The plates were incubated at 30 °C for 192 h and checked for the size of the colonies. The size of the colonies were measured manually and the average of ten colonies used to determine the average size.

### MNNG mutagenesis

**Fig. 1:**
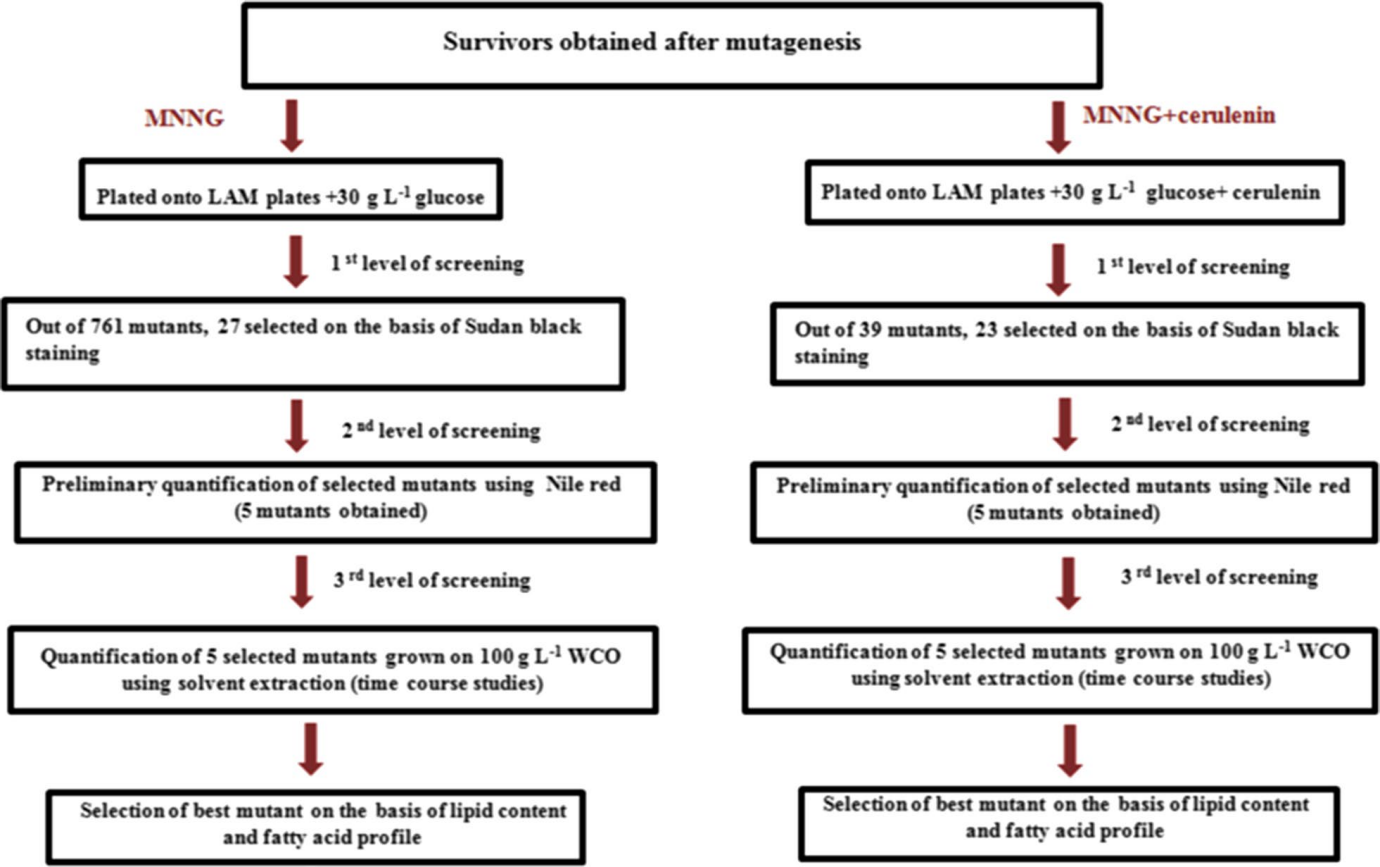
A schematic representation of the strategy followed to obtain the mutants

The overall scheme for mutagenesis and the experimental set up is shown in Fig. 1.

MNNG stock solution (10 mg/mL) was prepared in 95% (w/v) ethanol. An initial count of 2 × 10^8^ cells/mL was taken. For the cell viability experiments, the concentration of MNNG was varied from 0 to 500 µg/mL, with a fixed exposure time of 15 min. Next, keeping the MNNG concentration fixed, the exposure time of the cells was varied from 0 to 60 min. For both the experiments, the cells were incubated at 30 °C for specified time intervals in a shaking water bath. The action of MNNG was stopped by addition of 1 mL of filter sterilized, sodium thiosulphate (10%, w/v). The cells were centrifuged at 8000 rpm, 10 min and washed twice with distilled water. Different dilutions of the cells were made and plated onto agar plates on LAM media. Additionally another set of LAM media plates supplemented with cerulenin were incubated for 192 h at 30 °C to allow for growth of colonies and estimation of colony diameter.

### Preliminary screening of mutants by Sudan Black B staining

The Sudan Black B solution (SBB) was prepared by dissolving 0.08% (w/v) Sudan Black B in 95% (w/v) ethanol. Each individual colony was picked, transferred on a filter paper grid, stained with SBB solution and destained with 95% (w/v) ethanol as per Evans et al. [18].

### Secondary screening of mutants and lipid content by Nile red

The selected mutant colonies were inoculated into LAM (10 mL) at a cell count of 2 × 10^8^/mL and incubated on a rotary incubator shaker (120 rpm) at 30 °C for 120 h. Nile red (0.1 mg/mL) dissolved in acetone was used to estimate intracellular lipid content as per Liang et al. [19]. Aliquots (400 μL) were taken from each sample at 96 h for estimating the total lipid yield.

### Time course studies of the mutants in shake flasks

An initial pre-inoculum was grown in the liquid MGYP medium. The mutants demonstrating higher total lipid yield as quantitated by Nile red spectrofluorimetry were grown in LAM medium containing WCO (100 g L^−1^) instead of glucose as mentioned above. Samples were collected at 24 h intervals, for determination of biomass (g L^−1^), total lipid yield (g L^−1^) and lipid content (%).

### Stability of the mutants

The stability studies on the mutants were carried out by sub-culturing each mutant every month on the MGYP agar medium and checked for their lipid content up to 24 months. The 6th, 12th, 18th and 24th subcultures of the selected mutants were inoculated in MGYP broth and then into LAM medium with 100 g L^−1^ WCO as described earlier. The lipid content (%) was determined as mentioned under analytical methods.

### Molecular typing of mutants

The mutants were identified using molecular typing methods. Yeast genomic DNA was isolated using Qiagen DNeasy Kit following manufacturer’s instructions. Fungal LSU (large ribosomal subunit) and ITS (internal transcribed spacer) regions were amplified using standard PCR reaction [20]. The primers for the ITS1 (5′-TCCGTAGGTGAACCTGCGG-3′) and ITS4 regions (5′-TCCTCCGCTTATTGATATGC-3′) were used in the PCR amplification. For the LSU region, the primer LR7 (5′-TACTACCACCAAGATCT-3′) and 5.8 SR (5′-TCGATGAAGAACGCAGC-3′) were used to amplify the fragment. After amplification, the products were purified by using a geneO-spin PCR product Purification kit (GeneOmbio Technologies, Pune, India) and were directly sequenced using an ABI PRISM BigDye Terminator V3.1 kit (Applied Biosystems, USA). DNA sequencing was performed using primers used for PCR of LSU and ITS regions. The sequences were analyzed using Sequencing Analysis 5.2 software. BLAST analysis was performed at BlastN site at NCBI server (http://www.ncbi.nlm.nih.gov/BLAST).

### Analytical methods

#### Nile red fluorescence microscopy and spectrofluorimetry

The lipid accumulation was demonstrated by Nile red fluorescence microscopy. Both bright field and fluorescence microscopy were performed to check lipid accumulation of the mutants. Under an oil immersion lens, the yeast cells were observed with the help of a Zeiss microscope (Axio Scope A1) equipped with a digital camera. The images were captured using ProgRes CapturePro 2.7 software (Jenoptik Optical Systems, USA).

For quantitative lipid estimation by Nile red spectrofluorimetry, an aliquot (125 µL) of the cell suspension was added to 2.5 mL of 50 mM potassium phosphate buffer (pH 7.0). Cell samples without Nile red and controls (Nile red without cells) were also taken. To the samples, 12.5 µL of Nile red in acetone (0.1 mg/mL) was added and mixed well. From this, 250 µL was taken and loaded onto 96-well black plate and incubated in dark for 10 min. The wavelength used for excitation (λ_ex_) was 520 nm. The emission spectra were recorded in the wavelength of 530–750 nm on a spectrofluorimeter (SpectraMax M5, Molecular devices, CA, USA). The peak of the spectrum, which corresponded to the maximum fluorescence (λ_em_) was recorded. The triacylglycerol, triolein dissolved in acetone was used as the standard and a calibration curve was plotted and the total lipid yield estimated.

#### Biomass determination

The yeast biomass was harvested, made free of the residual substrate by successively washing the cells with hexane and methanol, oven-dried (50 °C, overnight) and the biomass estimated gravimetrically.

#### Determination of total lipid yield and lipid content

A modified protocol of Yu et al. [21] was used for lipid extraction. The WCO-free, oven-dried yeast cells (0.5 g) was lysed by acid treatment (4 M HCl) and kept on a shaking water bath (Medica instruments, Medica Industries, India) at 80 °C for 2 h. The acid hydrolyzed biomass was mixed with 20 mL of chloroform and methanol (1:1, v/v) and kept on a shaker at 30 °C for 3 h. The lipid extract was washed with 0.88% KCl (v/v) solution, the lower phase filtered and vacuum evaporated. The total lipid yield was estimated gravimetrically and the lipid content was also determined using the dry weight.

#### Fatty acid profiling of mutants

The total lipid obtained was transesterified as per Leung et al. [22]. Briefly, NaOH (1.5–3%, w/w of the lipid) was added in the presence of excess methanol. The solution was refluxed for 90 min at 60 °C and then the methanol was removed by vacuum evaporation to obtain crude fatty acid methyl ester (FAME). Chloroform: methanol (2:1) was added to reconstitute the crude FAME, which was then made free of water soluble impurities by successive washes with distilled water. The lower organic phase was aspirated, dried using anhydrous sodium sulphate and vacuum evaporated. The purified FAME was again reconstituted in chloroform: methanol (2:1, v/v) and used to determine the fatty acid profile and also for fuel properties.

A gas chromatograph (GC-2014, Shimadzu Corporation, Japan) with an FID detector equipped with an Rtx-2560 column (100 m length, 0.25 mm ID, Restek corporation, Bellefonte, USA) was used to determine the profile of the fatty acid methyl esters using the AOCS method [17]. The individual fatty acid methyl esters (FAMEs) were quantified by comparing their retention times with a standard FAME mix (i.e., a 37 component FAME mix, Supelco, USA).

#### Fuel properties of biodiesel of mutants grown on WCO

The various physico-chemical properties of the FAMEs or biodiesel from the mutants showing maximum lipid content were determined using predictive models and mathematical equations for the transesterified SCOs [9]. Density, saponification number (SN) and iodine value (IV) were determined both experimentally and predicted using mathematical equations. Other properties like cetane number (CN), kinematic viscosity were estimated using predictive equations [23]. The free fatty acid content (FFA), total acid number (TAN) and water content were determined using the Kittiwake DIGI biodiesel test kit (Kittiwake Developments Ltd., UK). The copper strip corrosion test was done as per the test specifications of ASTM D130.

#### Statistical analysis

All values are means of three independent experiments. The data obtained for biomass, total lipid yield and lipid content were from three independent determinations. The values obtained represent the mean ± standard deviation. The statistical analysis (One way ANOVA, Tukey–Kramer multiple comparison test) were done using GraphPad InStat ver 3.10 software and p < 0.05 were considered significant.

## Results and discussion

*Yarrowia lipolytica*, a model organism for lipid accumulation is known to accumulate SCOs as triacylglycerols (TAGs) and its metabolism is well adapted for oil utilization [24]. Its major drawback is its mediocre lipid content (27–57.8%) as reported for *Y. lipolytica* grown on industrial fats and WCO supplemented with glucose [4, 10]. UV has been used to increase lipid production from 45.4 to 58.9% for oleaginous yeasts like *Lipomyces starkeyi* DSM 70296 [14]. However, photoreactivation and reversion of mutants is a common feature associated with UV mutants and obtaining stable mutants is a challenge [25]. Chemical mutagens, namely, EMS and MNNG are alkylating agents that cause transition and transversions of chromosomal DNA, which is more genetically stable than the point mutations associated with UV treatments [26]. Chemical mutagenesis using EMS to enhance SCO content for biodiesel production has been previously used for *Y*. *lipolytica* [15]. Herein, a different alkylating agent, MNNG was used.

### MNNG mutagenesis of *Y. lipolytica* 3589

The tropical marine yeast, *Y. lipolytica* NCIM 3589 yielded a maximum lipid/biomass (Y_L/X_) yield coefficient of 0.43–0.45 g g^−1^ in 72–96 h when grown on 100 g L^−1^ WCO and was subjected to chemical mutagenesis by MNNG.

Choosing an optimal dose of the mutagen is very important as too high a dose could cause cell death. However an optimal dose (concentration and exposure time of the mutagen) can lead to an increase in the desired product along with stable mutants [27]. The dose response of the yeast cells to the concentration of the mutagen and to the time of exposure was carried out. For this, the survival curve of the yeast cells in response to varying concentrations of MNNG (0–500 μg/mL) at a fixed exposure time of 15 min was plotted in Additional file 1: Figure S1a. An exponential decrease in the number of colonies was seen as the concentration of MNNG was increased. MNNG at 500 μg/mL was found to have an inhibitory effect on the cell growth (99% mortality). Half the number of cells survived on exposure to MNNG (100 µg/mL) for 15 min at room temperature and this was considered as the LD_50_ value.

In the next experiment, to cross-check the effect of exposure time of the cells to MNNG, the concentration of 100 μg/mL of MNNG was fixed and the cells were exposed to MNNG at varying time intervals of 0–60 min. The survival curve of the yeast cells in response to varying exposure time to MNNG was plotted in Additional file 1: Figure S1b. Half the number of cells survived at an exposure time of 14 min of the mutagen and was considered as the LD_50_ value [27]. Hence, based on the results obtained as above, a concentration of 100 µg/mL MNNG and an exposure time of 15 min was used wherein a total of 761 mutants were generated. These mutants were screened to identify high SCO producers.

Finogenova et al. [28] have reported mutants of *Y. lipolytica* VKM Y-2373 with 43.9% increased ability as compared to the wild type strain to synthesize citric acid from glucose by using UV irradiation (4–6 min) and MNNG (40–80 µg/mL). A survival rate of below 25% was obtained for the concentration of 50 µg/mL of MNNG at a UV exposure time of 3–6 min. In contrast, a survival percentage of 10.9 was seen for *Y. lipolytica* 57 at a concentration of 500 µg/mL EMS for citric acid production [29]. In the yeast *Cryptococcus curvatus* ATCC 20509, EMS has been used to generate fatty acid auxotrophs (UfaM3) defective in the conversion of stearic acid to oleic acid [30] while MNNG has been used to increase lipase production in *Y. lipolytica* CBS 6303 [31].

Another strain *Y. lipolytica* DSM3286 was subjected to mutation using EMS and UV light to obtain mutants producing 10.5 fold higher levels of extracellular lipase than wild type strain using methyl oleate as substrate [32]. In another study, mutant LgX64.81 obtained by chemical mutagenesis of *Y. lipolytica* CBS6303 using EMS, had the highest potential for extracellular lipase production [31]. In *Y. lipolytica* NRRL YB-567, > 70, 90 and 99% mortality was observed in 2, 4 and 6 h respectively on UV irradiation [33]. Thus, in this study mutants from *Y. lipolytica* 3589 were isolated using a chemical mutagen (MNNG) at a low concentration of 100 µg/mL with an exposure time of 15 min.

### Effect of cerulenin on colony size

Cerulenin ((2S) (3R) 2, 3-epoxy-4-oxo-7, 10 dodecadienoylamide)), a fatty acid synthase inhibitor, was originally isolated from the culture broth of the fungus *Cephalosporium caerulens*. It inhibits the crucial condensation step of fatty acid synthesis by binding to the cysteine-SH in the active site of the condensing enzyme component (β-keto-acyl-ACP synthase) of the yeast fatty acid synthase. This prevents the condensation of acetyl-CoA and malonyl-CoA to form β-keto-acyl-ACP, an important step in fatty acid biosynthesis [34].

Incorporation of this inhibitor into the growth medium would inhibit the growth of the wild type cells as demonstrated by a decrease in the colony size. Mutants which possess high fatty acid synthase activity would be capable of overcoming this inhibition and be unaffected in their growth by this inhibitor. Based on this assumption, Wang et al. [35] isolated high lipid producing mutants of the yeast *Rhodotorula glutinis* with an increase in lipid content from 18.2% in control (wild type) to 28.8–30.7% in mutants. Thus, in this study high SCO yielding mutants were obtained via two approaches—by non-specific mutagenesis by MNNG alone and by using an additional selective pressure with cerulenin (MNNG + cerulenin). Those high lipid yielding colonies capable of overcoming the inhibition exerted by cerulenin on fatty acid synthase would be identifiable based on their colony size, which would be larger than the low lipid producing ones [35].

Studies on the wild type yeast strain showed a visible decrease in colony size upon increasing cerulenin concentrations. The colony size decreased from 2.2 mm in control to 1.1 and < 0.5 mm for the wild type strain treated with 15 and 20 µg/mL cerulenin, respectively. Thus, 15 µg/mL was chosen for further studies as a 50% decrease in the colony size was observed. Mutants were selected with a colony size greater than 1.1 mm. In the MNNG + cerulenin plates, 39 colonies were selected with a colony size > 1.1 mm and further screened using Sudan Black B staining (Fig. 1).

Tapia et al. [14] grew *Lipomyces starkeyi* DSM 40296 in presence of 10 µg/µL cerulenin after UV mutagenesis and found that the colony size decreased from 0.7 mm for high lipid producers to 0.2 mm for low lipid producers. In *Rhodotorula glutinis* AY 91015, 33 colonies with diameter > 2 mm were selected as high lipid producers amongst other low lipid producing colonies with lesser diameter after irradiation with carbon ions (80 MeV/u energy) and 8.96 µmol/L cerulenin [35].

### Sudan Black B staining of the colonies

Sudan Black B (SBB) is a dye belonging to the phenyl-azo-naphthyl-azo-naphthyl type which stains lipids [36]. A rapid, replica-printing method using SBB was used as a preliminarily screen for high lipid producing colonies [18]. From the MNNG treated cells, a total of 761 mutants were screened and 27 selected based on the SBB staining method. For the MNNG + cerulenin plates, of the 39 colonies screened, 23 colonies were selected (Fig. 1). All these colonies showed an intense dark blue colour when compared to the wild type strain.

Sudan Black B has been used to screen 129 yeasts isolated from different Brazilian regions for their oleaginous potential [37]. In another study, *Cryptococcus podzolicus*, *Trichosporon porosum* and *Pichia segobiensis* were selected as potential lipid producers using the same technique [38]. Similarly, Lindquist et al. [33] showed that the mutant strains of *Y. lipolytica* NRRL YB-567 when subjected to UV irradiation showed a 48% increase in oil production over the wild type strain based on SBB densitometry.

Thus, in the present study, using both the approaches i.e., MNNG and MNNG + cerulenin, a total of 50 colonies were selected using the preliminary SBB staining method.

### Estimation of neutral lipid by Nile red spectrofluorimetry

The fluorescent dye, Nile red has been reported to stain intracellular lipid droplets that can be observed using fluorescent microscopy [39]. It has been previously used to develop a high throughput assay to quantify total lipids or TAG fraction for large-scale screening of yeast samples [40]. Liang et al. [19] have earlier quantified the neutral lipid content in microbes using this fluorescent dye. In this study, a calibration curve using the triacylglycerol triolein as standard was performed and the total lipid yield in the selected mutants determined. Table 1 indicates the amount of lipid accumulated in the mutants grown on LAM containing 30 g L^−1^ glucose at 96 h. Of the above selected 50 mutants, 10 mutants gave a higher total lipid yield (0.24–0.39 g L^−1^) as compared to the wild type (0.20 g L^−1^). The mutants-YlC4, YlB2 and YlB3 (0.18–0.19 g L^−1^) were also selected which showed comparable lipid content as the wild type. The mutants obtained after MNNG treatment were designated as—YlB6, YlC4, YlC5, YlC6 and YlC7 while those after MNNG + cerulenin treatment were designated as—YlB2, YlB3, YlD5, YlE1 and YlE2. Amongst the mutants, YlE1 and YlC7 exhibited maximum total lipid yield of 0.39 and 0.30 g L^−1^, respectively.

**Table 1:** Estimation of total lipid yield of the mutants using Nile red fluorimetry

Treatment	Mutant	Total lipid yield (g L^−1^)
MNNG	YlB6	0.24 ± 0.01
	YIC4	0.19 ± 0.01
	YIC5	0.24 ± 0.01
	YIC6	0.27 ± 0.01
	YIC7	0.30 ± 0.02
	YIB2	0.19 ± 0.01
MNNG + cerulenin	YIB3	0.18 ± 0.01
	YID5	0.27 ± 0.02
	YIE1	0.39 ± 0.02
	YIE2	0.20 ± 0.01
	WT	0.20 ± 0.01

The mutants were grown on LAM containing 30 g L^−1^ glucose for 96 h. The values represent the mean ± SD of three independent determinations

### Time course studies of the mutants producing higher lipid on WCO

**Fig. 2:**
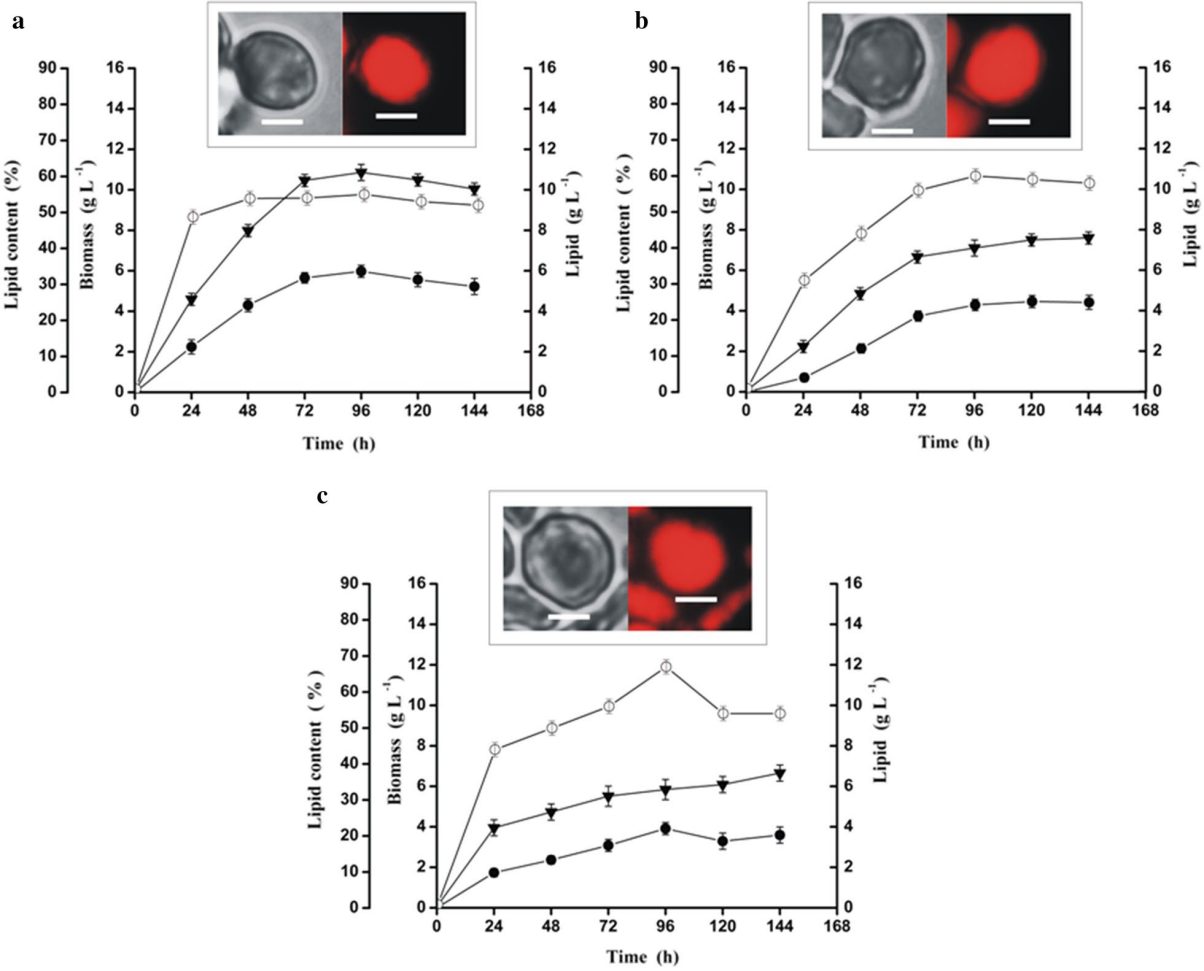
Time course studies to determine biomass, total lipid yield and lipid content of MNNG and MNNG + cerulenin treated mutants. Mutants **a** YlB6, **b** YlC7 and **c** YlE1were grown on 100 g L^−1^ WCO as mentioned in “Methods”. All values are represented as mean ± SD, determined after three independent experiments. Biomass (g L^−1^)—black down pointing triangle, Total lipid yield (g L^−1^)—black circle, Lipid content (%)—white circle. Inset: In each graph light microscopy (left panel) and Nile red fluorescence microscopy (right panel) images of the respective *Y. lipolytica* mutants under 100 × oil immersion objective. Bar indicates 4 μm

The above selected 10 mutants were evaluated in terms of biomass, total lipid yield and lipid content by growing them in LAM containing 100 g L^−1^ WCO. The results of the time course studies of the highest two SCO yielding mutants (YlB6 and YlC7) obtained after MNNG treatment are presented in Fig. 2a, b. The other three mutants (YlC4, YlC5 and YlC6) obtained after MNNG treatment are presented in Additional file 2: Figure S2. Time course studies of MNNG + cerulenin treated mutants are presented in Fig. 2c. The other four mutants obtained after MNNG + cerulenin treatment (YlB2, YlB3, YlD5and YlE2) are presented in Additional file 3: Figure S3. The results indicate that biomass (X, g L^−1^) of mutants treated with MNNG varied from 2.24 to 10.86 g L^−1^. The total lipid yield (L, g L^−1^) varied from 0.69 to 5.67 g L^−1^. The lipid content (%) also varied from 24% at 24 h for mutant YlC6 to a maximum of 60% for the mutant YlC7 at 96 h.

For the MNNG + cerulenin treated mutants (Fig. 2c and Additional file 3: Figure S3a, b, c, d), the biomass varied from 2.3 to 8.98 g L^−1^. The total lipid yield varied from 0.84 to 5.1 g L^−1^. The lipid content was lowest at 21% at 24 h in the mutant YlB3 and was maximum at 67% in 96 h for the mutant YlE1. From Fig. 2, Additional file 2: Figure S2 and Additional file 3: Figure S3, it can be seen that the lipid body in the yeast cells (by bright field microscopy) could be visualized as revealed in the Nile red fluorescence images (insets).

Three mutants enriched for lipid contents were obtained from either one of these treatments, viz., YlB6 and YlC7 obtained after MNNG treatment and YlE1 after MNNG + cerulenin treatment. The mutants YlB6 and YlC7 had 55 and 60% lipid contents, respectively, after 96 h compared to 45% for the wild type, which corresponds to a 1.22 and 1.33-fold improvement. The mutant YIE1 had a lipid content of 67% after 96 h, which is a 1.49-fold improvement compared to wild type.

Table 2 describes the X (biomass produced), L (total lipid yield obtained), Y_L/X_ (lipid/biomass yield coefficient), Q_X_ (volumetric biomass productivity), Q_L_ (volumetric lipid productivity) of the mutants grown on WCO for 96 h. The biomass varied from 4.4 to0 10.68 g L^−1^. The total lipid yield varied from 1.57 to 5.97 g L^−1^. The lipid/biomass yield coefficient also varied from 0.35 to 0.67 g g^−1^. When compared to the wild type, maximum biomass and total lipid yield was obtained from the MNNG treated mutant YIB6 (10.86 and 5.97 g L^−1^, respectively). The volumetric biomass productivity, Q_x_ (g L^−1^ h^−1^) for all the mutants varied from 0.045 to 0.113 g L^−1^ h^−1^ and was the highest for the mutant YIB6 with a value of 0.113 g L^−1^ h^−1^. The volumetric lipid productivity, Q_L_ (g L^−1^ h^−1^), an important parameter varied from 0.016 to 0.062 g L^−1^ h^−1^ for all the mutants. The volumetric lipid productivity reached the maximum in the mutant YIB6 (0.062 g L^−1^ h^−1^) followed by YlC7 with 0.044 g L^−1^ h^−1^ and the MNNG + cerulenin treated mutant YlE1 with 0.041 g L^−1^ h^−1^.

**Table 2:** Biomass, total lipid yield, yield coefficient, volumetric productivities of biomass and lipid of mutants

Treatment	Mutant	X (g L^−1^)	L (g L^−1^)	Y_L/X_ (g g^−1^)	Q_X_ (g L^−1^ h^−1^)	Q_L_(g L^−1^ h^−1^)
No treatment	WT	7.1 ± 0.03^a^	3.2 ± 0.03^a^	0.45 ± 0.004^a,d^	0.074 ± 0.0002^a^	0.033 ± 0.0002^a^
	YIB6	10.86 ± 0.01^b^	5.97 ± 0.01^b^	0.55 ± 0.001^b^	0.113 ± 0.0001^b^	0.062 ± 0.0001^b^
MNNG	YIC4	5.76 ± 0.03^c^	2.73 ± 0.03^c^	0.47 ± 0.005^c,d^	0.059 ± 0.0003^c^	0.028 ± 0.0003^c^
	YIC5	5.91 ± 0.02^d^	2.74 ± 0.02^c^	0.46 ± 0.004^d^	0.062 ± 0.0002^d^	0.028 ± 0.0002^c^
	YIC6	6.25 ± 0.03^e^	2.23 ± 0.03^d^	0.35 ± 0.005^e^	0.065 ± 0.0002^e^	0.023 ± 0.0003^d^
	YIC7	7.1 ± 0.03^a^	4.28 ± 0.01^e^	0.60 ± 0.004^f^	0.073 ± 0.0002^a^	0.044 ± 0.0002^e^
	YIB2	7.0 ± 0.03^a^	3.7 ± 0.02^f^	0.53 ± 0.006^g^	0.072 ± 0.0006^a^	0.038 ± 0.0001^f^
	YIB3	5.40 ± 0.03^f^	1.92 ± 0.03^g^	0.35 ± 0.006^e^	0.056 ± 0.0003^f^	0.019 ± 0.0003^g^
MNNG + cerulenin	YID5	5.73 ± 0.01^c^	2.42 ± 0.02^h^	0.42 ± 0.006^h^	0.059 ± 0.0001^c^	0.025 ± 0.0002^h^
	YIE1	5.84 ± 0.03^d^	3.91 ± 0.01^i^	0.67 ± 0.004^i^	0.061 ± 0.0003^d^	0.041 ± 0.0001^i^
	YIE2	4.4 ± 0.03^g^	1.57 ± 0.02^j^	0.35 ± 0.004^e^	0.045 ± 0.0002^g^	0.016 ± 0.0002^j^

The values indicate the mean ± standard deviation (n = 3). Mean values within a column with different superscript letters (^a,b,c,d,e,f,g,h,i,j^) differ significantly and were determined statistically using Graph Pad InStat ver 3.10 software (One-way ANOVA, Tukey–Kramer multiple comparison test, p < 0.05). Separate analysis was done for each column. The wild type and mutants were grown on LAM containing 100 g L^−1^ WCO for 96 h. *WT* wild type, *X* biomass, *L* total lipid yield, *Y*_*L/X*_ lipid/biomass yield coefficient, *Q*_*X*_ volumetric biomass productivity, *Q*_*L*_ volumetric lipid productivity. All yields were determined gravimetrically

The statistical analysis (one way ANOVA and Tukey–Kramer multiple comparison test) further proved that the differences between wild type and mutants were significant (Table 2). For biomass, the wild type, YlB2 and YlC7 belonged to the same group while YlB6 and YlE1 belonged to separate groups. The lipid yields of all mutants and the wild type differed and were separately grouped. For Y_L/X_, notably, YlB6, YlC7 and YlE1 showed significantly higher values and were separately grouped. For Q_X_, the wild type, YlC7 and YlB2 belonged to the same group. For Q_L_, the wild type, YlB6, YlC7 and YlE1 belonged to different groups and were again significantly higher as compared to the wild type.

On the basis of the results obtained from the MNNG treatment, YlB6 was selected because its cultures showed the maximum biomass, total lipid yield and volumetric biomass and lipid productivities. The mutant YlC7 was also selected because it showed a lipid content of 60% and an increased lipid productivity as compared to the wild type. From the MNNG + cerulenin treatment, the mutant YlE1 was selected which showed the maximum lipid content (67%) along with increased volumetric lipid productivity as compared to the wild type.

The lipid contents in the mutants YlB6, YlC7 and YlE1 were comparable to the mutant of *Colletotrichum* sp. DM06, wherein a ~ 1.7-fold increase in lipid content was observed after its genetic transformation with the *CtDGAT2b* gene from *Candida tropicalis* SY005 [41]. The lipid content of the two mutants (YlC7 and YlE1) studied in this study were higher than the six mutants of *Lipomyces starkeyi* reported with highest lipid content of 58.9% after UV mutagenesis and cerulenin treatment [14]. In another study, after physical mutagenesis of *Rhodosporidium toruloides* np11 using atmospheric and room temperature plasma method and chemical mutagenesis with MNNG, the mutant strain *R. toruloides* XR-2, accumulated 41% lipids as compared to 23% of the wild type [16] which was much lower as compared to the results obtained in this study.

A number of studies have been carried out for lipid production by metabolically engineered *Y. lipolytica* strains grown mainly on glucose as carbon source. Blaczeck et al. [42] rewired the native metabolism through combinatorial multiplexing and phenotypic induction and obtained strains with high lipid content (90%) and lipid titers (25 g L^−1^). Liu et al. [15] employed an evolutionary engineering approach along with a floating cell enrichment process to obtain a high lipid titers of 39.1 g L^−1^ with lipid content (87%) and 0.243 g g^−1^ yield, and 0.56 g L^−1^ h^−1^ average specific productivity [15]. Qiao et al. [43] obtained a phenotype capable of accumulating high yield (0.243 g g^−1^), titer (55 g L^−1^) and productivity (0.71 g L^−1^ h^−1^) by overexpressing delta-9 stearoyl-CoA desaturase, Acetyl-CoA carboxylase (ACC1), and Diacylglyceride acyl-transferase (DGA1). Another study by Tai and Stephanopoulos [11] in which a strain simultaneously overexpressing a tandem gene construct of acetyl CoA carboxylase (ACC1) and diacylglycerol acyltransferase (DGA1) grown in a 2 L bioreactor resulted in 61.7% lipid content [11], similar to that reported here. The lipid content of the mutants in the present study were comparable to the mutants accumulating 65–75% lipid content obtained by deleting the genes responsible for β-oxidation and producing mutations in the G3-P shuttle pathway [44]. Though the yeast cannot utilize lignocellulosic biomass or starch, it has been engineered the yeast to grow on different substrates [45]. For example, an obese strain, *Y. lipolytica* XYL + Obese was generated by overexpression of GPD1 and DGA2 involved in TAG synthesis and XR (xylose reductase), XDH (xylitol dehydrogenase) and XK (xylulose kinase) required for growth on xylose while lipid degradation has been eliminated through deletion of the POX and TGL4 genes. The strain is able to accumulate high levels of lipids (36.5%) when grown on yeast nitrogen base—xylose medium similar to that observed when grown on fructose in shake flask cultures [46].

In this study, classical mutagenesis was tried in order to increase the lipid content of *Y. lipolytica* 3589 for biodiesel production. The mutants were preliminarily screened on glucose to identify high lipid yielding mutants which were then checked for lipid accumulation on WCO, as it is known that a low cost substrate can reduce production costs.

Our studies to obtain strains with increased SCO content suitable for biodiesel production resulted in isolation of mutants with capacity to accumulate lipid up to 67% using a classical and proven method for strain improvement.

### Determination of the fatty acid profile of the selected mutants

The three mutants, YlB6, YlC7 and YlE1 were evaluated for their fatty acid profile in order to check if their lipids were suitable for use as a feedstock for biodiesel production. Table 3 lists the fatty acid profiles of the three mutants obtained in comparison with the parental yeast strain. The fatty acid profile showed the presence of C16 and C18 saturated fatty acids (SFAs) and monounsaturated fatty acids (MUFAs). YlC7 showed a lower content (39.80%) whereas YlB6 and YlE1showed a higher content (54.1 and 58.1%, respectively) of total SFAs in comparison to the wild type (56.2%). Both palmitic and stearic acid were higher in YlB6 (29.1 and 6.3%), YlC7 (28.1 and 6.1%) and YlE1 (30.4 and 8.5%) as compared to the wild type *Y. lipolytica* NCIM 3589 (21.1 and 3.4%) as shown in the table.

**Table 3:** Comparison of fatty acid methyl ester profile of the mutants with the wild type

Fatty acid methyl ester	(wt % of total fatty acid methyl esters)			
	YlB6	YIC7	YIE1	WT^a^
Caprylic acid methyl ester (C8:0)	ND	ND	ND	25.0
Lauric acid methyl ester (C12:0)	ND	ND	ND	3.2
Myristic acid methyl ester (C14:0)	ND	ND	ND	1.7
Palmitic acid methyl ester (C16:0)	29.1	28.1	30.4	21.1
Stearic acid methyl ester (C18:0)	6.3	6.1	8.5	3.4
Arachidic acid methyl ester (C20:0)	9.8	2.7	8.7	ND
Heneicosanoic acid methyl ester (C21:0)	8.9	2.9	10.5	1.8
Total of fatty acids: Saturated	54.1	39.8	58.1	56.2
Palmitoleic acid methyl ester (C16:1)	ND	4.1	10.0	0.9
cis-10-Heptadecanoic acid (C17:1)	ND	ND	ND	8.0
Oleic acid methyl ester (C18:1n9c)	33.6	40.1	25.4	21.0
cis-11Eicosanoic acid (C20:1)	ND	ND	ND	2.0
Total of fatty acids: Monounsaturated	33.6	44.2	35.3	31.9
Linoleic acid methyl ester (C18:2n6c)	12.3	16.0	6.6	11.8
Total of fatty acids: Polyunsaturated	12.3	16.0	6.6	11.8
Total of fatty acids	100	100	100	99.98

The wild type and mutants were grown on LAM containing 100 g L^−1^ WCO. The values represent the mean ± SD of three independent determinations

*WT* Wild type; ^a^as reported earlier [9], *ND* Not detected

The total MUFAs were higher than the wild type (31.9%) in all the three mutants, at 33.6, 44.2 and 35.4% in YlB6, YlC7 and YlE1, respectively. Palmitoleic acid (C16:1) was present in significantly higher amounts in YlC7and YlE1 (4.1 and 10.0%) as compared to the wild type (0.9%). The content of desirable oleic acid (C18:1n9c) was also higher in all the three mutants-YlB6 (33.6%), YlC7 (40.1%) and YlE1 (25.4%) as compared to the wild type (21.0%). Linoleic acid (C18:2) was higher in YlB6 (12.3%) and YlC7 (16.0%) whereas it was lower in YlE1 (6.6%) when compared to the wild type (11.8%).

The three mutants possessed higher amounts of total C16 and C18 SFAs (35.4, 34.2 and 38.9% for YlB6, YlC7and YlE1, respectively) as compared to 24.5% of the wild type. The total of C16 and C18 MUFAs of the three mutants were also higher (33.6, 44.2 and 35.4% for YlB6, YlC7and YlE1, respectively) in comparison with the wild type (21.9%). The fatty acid profiles of the three mutants were hence found suitable for biodiesel production.

The fatty acid profile of *Rhodosporidium toruloides* XR-2 mutant contained myristic acid (2.6%), palmitic acid (36.7%), palmitoleic acid (3.1%), stearic acid (11.5%), oleic acid (36.4%) and linoleic acid (2.8%) [16] which was comparable to the fatty acid profile of the three mutants in the present study. The microalga *Nannochloropsis* sp. was mutated using EMS to yield two mutants, LARB-202-2 and LARB-202-3 with palmitic acid (C16:0, 52.5–53.7%), myristic acid (C14:0, 9.5–8.3%), lesser proportion of oleic acid (C18:1n9c, 15.81-16.43%) and higher proportion of the undesirable eicosapentanoic acid (C20:5, 11.22–12.54%) and was therefore not suitable for biodiesel production [47].

In *Y. lipolytica*, lipid accumulation occurs via two pathways: the de novo pathway that occurs in presence of non-hydrophobic substrates and the ex novo pathway that occurs in presence of hydrophobic substrates [48]. For screening, the mutants were grown on glucose which would have promoted *de novo* lipid accumulation. The lipid yield on glucose was found to be low (0.2–0.4 g L^−1^). In the next step, the mutants were grown on WCO, a hydrophobic substrate, which resulted in lipid accumulation by the ex novo pathway. As the results indicate, the lipid yield (1.57–5.97 g L^−1^) increased and the mutants could utilize this substrate for increased lipid accumulation. *Y. lipolytica* is known to either incorporate fatty substrates directly in an unchanged manner or accumulate the substrate in a modified form [48]. In this case, the mutants and wild type both had fatty acid compositions modified from the feed oil. It can be seen from Table 3 that the levels of palmitic acid (40.5%), oleic acid (C18:1, 40.39%) and linoleic acid (C18:2, 10.35%) contents in the WCO were altered in the SCOs of the wild type strain and mutants YlC7 and YlE1. Additionally, SFAs like stearic acid, arachidic acid and heneicosanoic acid and MUFAs like palmitoleic acid (C16:1) could be detected.

### Determination of fuel properties of the FAMEs or biodiesel from the selected mutants

The fuel properties of the selected mutants, YlB6, YlC7 and YlE1 were determined experimentally and using different mixing rules and prediction equations. Table 4 summarizes the different fuel properties. The experimental and predicted values of density were comparable for the mutants and the wild type. The experimental values of water content, FFA and copper strip corrosion test were also comparable for both mutants and wild type strain. The TAN was lower in the three mutants (2 mg NaOH g^−1^ for all three) as compared to the wild type (2.8 mg NaOH g^−1^). An important observation was that for all the three mutants, YlB6, YIC7 and YlE1, the cetane numbers (CN) (66.7, 59.43 and 68.6, respectively) were higher than the CN of the wild type (50.8). The CN of the three mutants was higher than the CN value of 54 reported for *Candida freyschussii* ATCC 18737 [49]. A higher CN helps ensure good cold-start properties and reduces smoke formation [50]. Biodiesel with high MUFA content has better characteristics with respect to ignition quality, nitrogen oxide emissions, fuel stability, and flow properties. Hence methyl esters of palmitoleic (C16:1) and oleic (C18:1) acids as seen in the FAME profiles of the mutants YlB6,YlC7 and YlE1 are warranted as they are liquid at room temperature. Thus, an ideal biodiesel is made primarily of monounsaturated with balanced levels of saturated and polyunsaturated methyl esters [51] as observed for FAME profiles of the mutants in this study. The HHV of all the three mutants (40.49–40.9 M J kg^−1^) was higher than that of the wild type (36.77 M J kg^−1^). HHV or gross calorific value is the amount of energy released upon combustion of 1 g of the fuel at initial temperature [52]. This meant that the FAMEs or biodiesel produced by the three mutants possessed more energy as compared to the wild type strain.

**Table 4:** Fuel properties of biodiesel produced by the mutants and wild type grown on WCO

Property/Test	Results for *Y. lipolytica* NCIM 3589				US biodiesel standards ASTM D6751	European biodiesel standards EN14214	Indian biodiesel standards IS15607
	YlB6	YIC7	YIE1	WT			
Density at 25 °C (g cm^−3^)^a^	1.07 (0.87)	1.07 (0.86)	1.08 (0.88)	1.04 (0.87)	NS	0.86-0.90	0.86-0.90
Water content (vol%)^a^	ND	ND	ND	ND	0.05 max	0.25 max	0.03 max
TAN (mg NaOH g^-1^)^a^	2	2	2	2.8	0.8 max	0.5 max	0.5 max
FFA (%)^a^	1	1	1	1.4	NS	NS	NS
Cu strip corrosion^a^	Class 1a	Class 1a	Class 1a	Class 1a	Class 3 max	Class 1 max	Class 1 max
CN^c^	66.7	59.43	68.6	50.8	47-65	51 min	51 min
Kinematic viscosity (40 °C; mm^2^ s^−1^)^c^	5.07	4.57	5.09	3.6	1.9-6.0	3.5-5.0	3.5-5.0
SN^a^	190.38 (194.65)	197.6(193.75)	197.4 (192.28)	256.16 (249.4)	NS	NS	NS
IV^a^	50.03 (47.9)	69.4 (65.82)	50.2 (42.58)	37.8 (47.9)	NS	120max	NS
HHV(M J kg^−1^)^b^	40.87	40.49	40.9	36.77	NS	NS	NS
Concentration of γ-linolenic acid (C18:3) (%)^a^	0	0	0	0	NS	12max	NS
FAME having ≥ 4 double bonds (%)^a^	ND	ND	ND	ND	NS	1 max	NS

The wild type and mutants were grown on LAM containing 100 g L^−1^ WCO. The experimental values are the mean ± SD of three independent determinations

*WT* Wild type, as reported earlier [9]; *ND* Not Detected, *NS* Not Specified

^a^Experimentally determined values, followed by predicted ones, if any, in brackets

^b^Calculated using predicted values of SN and IV

^c^Predicted values as mentioned in Materials and methods [23]

The other properties like density, kinematic viscosity, SN and IV were all found to lie within the limits specified by national and international specifications. Density is an important physical fuel property of biodiesel which depends on the fatty acid profile of alkyl esters present which in turn is affected by the raw materials used in fuel production. The densities of the mutants YlB6, YlC7 and YlE1 were 1.07, 1.07 and 1.08 g cm^−3^, respectively and are comparable to that of the wild type strain (1.04 g cm^−3^). The fuel viscosity is known to play a critical role in the fuel spray, mixture formation and combustion process. As the fatty acid chain length increases, viscosity increases while it is observed to decrease with an increase in unsaturation [51]. Kinematic viscosity was lower in the wild type strain (3.6 m m^2^ s^−1^) as compared to the three mutants (4.57–5.09 m m^2^ s^−1^) as the mutants possessed long chain SFAs like arachidic acid (C20:0) and heneicosanoic acid (C21:0). The density and viscosity of the mutants were also comparable to the density value obtained by transesterifying lipid from *Cryptococcus curvatus* ATCC 20508 grown on crude glycerol [53]. Iodine value (IV) which indicates the degree of unsaturation was higher in the mutants YlB6 -50.03 (47.9) [values in parentheses indicate the experimentally determined values], YIC7- 69.4 (65.82) and YlE1 50.2 (42.58) as compared to the wild type 37.8 (47.9) as the mutants possessed more proportion of MUFAs and PUFAs. Saponification numbers (SN), which indicate the free triglyceride content of the sample, were lower for all the three mutants as compared to the wild type. The SN, IV and HHV were also found to be similar to the values obtained from two fungi, *Alternaria* sp. and *Colletotrichum* sp. grown on glucose [54]. The IV and density of the three mutants were found to be similar to the values obtained from *Aspergillus* sp. grown on corncob waste liquor [55]. Thus, a good balance of SFAs and MUFAs was present in the mutants isolated by chemical mutagenesis in this study. This was reflected in the biodiesel properties of all the three mutants which were found to lie within the range specified by different biodiesel standards.

### Stability of the mutants

The three mutants, YlB6, YIC7 and YlE1 were subcultured every month 24 months and the lipid content was estimated for the 6th, 12th, 18th and the 24th subcultures. Table 5 shows the lipid content of the mutants after repeated sub-culturing. It was found that the lipid content of the mutants YlB6, YIC7 and YlE1 at 54.8, 59.6 and 66.8%, after the 24th subculture remained the same. This indicated that the mutants generated by chemical mutagenesis were stable with respect to lipid production for up to 2 years indicating the selected phenotypes are stable.

**Table 5:** Lipid content of the three mutants, YlB6, YlC7 and YlE1 after the 6th, 12th, 18th and 24th subculture

Number of the subculture	YlB6	YlC7	YlE1
6	55.1 ± 0.01	60.08 ± 0.02	67.2 ± 0.02
12	54.9 ± 0.02	60.05 ± 0.02	66.9 ± 0.02
18	54.8 ± 0.02	59.8 ± 0.02	66.8 ± 0.02
24	54.8 ± 0.02	59.6 ± 0.02	66.8 ± 0.02

### Molecular typing of the three selected mutants

Amplicons of size 1715 and 650 bp were obtained using the LSU and ITS region primers respectively for the strain YIB6. Amplicons of similar sizes were obtained for YlC7 and YIE1 using the LSU and ITS regions. The amplicon size was confirmed using reference ladder. The PCR and DNA sequence results indicate that all the three strains under study showed similarity to *Y. lipolytica* as indicated in the Additional file 4: Table S1. The LSU and ITS regions of all the three mutants- YlB6, YlC7 and YlE1 showed 100 and 99% sequence identity respectively to *Y. lipolytica*. This indicates that the three mutant strains that were generated were mutant varieties of the wild type *Y. lipolytica* strain used in the present study.

## Conclusions

The chemical mutagen, MNNG was used for the first time to generate genetically stable high lipid producing mutants from *Y. lipolytica* NCIM 3589. A total of 800 mutants (761 MNNG treated and 39 MNNG + cerulenin treated) for high SCO yields were screened using a two-stage selection with Sudan Black B and Nile Red. The additional selection pressure using cerulenin was carried out for the first time in *Y. lipolytica* to isolate high SCO yielding mutants. Three stable, high SCO yielding mutants of *Y. lipolytica* NCIM 3589, namely, YlB6 and YIC7 using MNNG and YlE1 using MNNG + cerulenin treatment (able to overcome fatty acid synthase inhibition) were selected with increased lipid content of 55, 60 and 67%, respectively. An increase in lipid productivities was demonstrated for YlB6, YlC7 and YlE1which were 1.87- , 1.33- and 1.24-fold, respectively as compared to the wild type. The mutants had a fatty acid profile consisting predominantly of desirable C16 and C18 SFAs and MUFAs, with the absence of undesirable PUFAs suggesting their suitability as good quality biodiesel. The experimentally determined and predicted fuel properties of the FAMEs from the mutants lie within the limits specified by the existing biodiesel standards. Thus, the results in this study suggest the potential of the SCO feedstock from the mutants obtained by chemical mutagenesis as biofactories for biodiesel production when grown on waste cooking oil and in turn may also help in the environmental management of waste cooking oil. Direct conversion of WCO often entails its pretreatment as it leads to soap formation resulting in low yield and poor quality of biodiesel. Many of these processes require high energy inputs, are chemical/enzyme/solvent intensive and require long time periods for conversion [5, 8, 22]. In fact Zhang et al. [56] have estimated the production cost of biodiesel directly from WCO using different processes with prices ranging from $644 to 884/tonne. This study is relevant as an alternative possibly low-cost strategy to increase SCO levels that could eventually pave the way for future large-scale production of biodiesel using oleaginous yeasts.

## Additional files

**Additional file 1: Figure S1.** The survival curve of *Y. lipolytica* NCIM 3589 in presence of MNNG. (a) different concentration of MNNG (0 - 500 µg/ml). (b) different exposure time to MNNG (0-60 min). (The dotted line indicates the LD _50_ value of 100 µg/ml and ~15 min).

**Additional file 2: Figure S2.**Time course studies to determine biomass, total lipid yield and lipid content of MNNG treated mutants. Mutants (a) YlC4, (b) YlC5 and (c) YlC6 were grown on 100 gL^−1^ WCO as mentioned in Methods. All values are represented as mean ± SD, determined after three independent experiments. Biomass (gL^−1^) -black down pointing triangle,Total lipid yield (gL^−1^) -black circle, Lipid content (%)-○. Inset: In each graph, light microscopy (left panel) and Nile red fluorescence microscopy (right panel) images of the respective *Y. lipolytica* mutants under 100 x oil immersion objective. Bar indicates 4 μm.

**Additional file 3: Figure S3.** Time course studies to determine biomass, total lipid yield and lipid content of MNNG + cerulenin treated mutants. Mutants (a) YlB2, (b) YlB3, (c) YlD5 and (d) YlE2 were grown on 100 gL^−1^ WCO as mentioned in Methods. All values are represented as mean ± SD, determined after three independent experiments. Biomass (gL^−1^) -black down pointing triangle, Total lipid yield (gL^−1^) - black circle, Lipid content (%) - ○. Inset: In each graph light microscopy (left panel) and Nile red fluorescence microscopy (right panel) images of the respective *Y. lipolytica* mutants under 100 × oil immersion objective. Bar indicates 4 μm.

**Additional file 4: Table S1.** Lipid content of the three mutants, YlB6, YlC7 and YlE1 after the 6th, 12th, 18th and 24th subculture.

## Abbreviations

WCO: waste cooking oil
(MNNG): 1-methyl-2-nitro-1-nitrosoguanidine
SCOs: single cell oils
TAGs: triacylglycerols
UV: ultraviolet light
EMS: ethyl methanesulfonate
MSTFA: N-(trimethylsilyl) trifluoroacetamide
TCMS: trimethylchlorosilane
LAM: lipid accumulation medium
LSU: large ribosomal subunit
ITS: internal transcribed spacer
FID: flame ionization detector
SFAs: saturated fatty acids
MUFAs: monounsaturated fatty acids
PUFAs: polyunsaturated fatty acids
SN: saponification number
IV: iodine value
FFA: free fatty acids
TAN: total acid number
HHV: higher heating value
CN: cetane number
