# Supplementary figures and images for: Mutants of Yarrowia lipolytica NCIM 3589 grown on waste cooking oil as a biofactory for biodiesel production
Source: Microb Cell Fact. 2017 Oct 24;16:176. doi: 10.1186/s12934-017-0790-x (PMC5655982; doi:10.1186/s12934-017-0790-x)

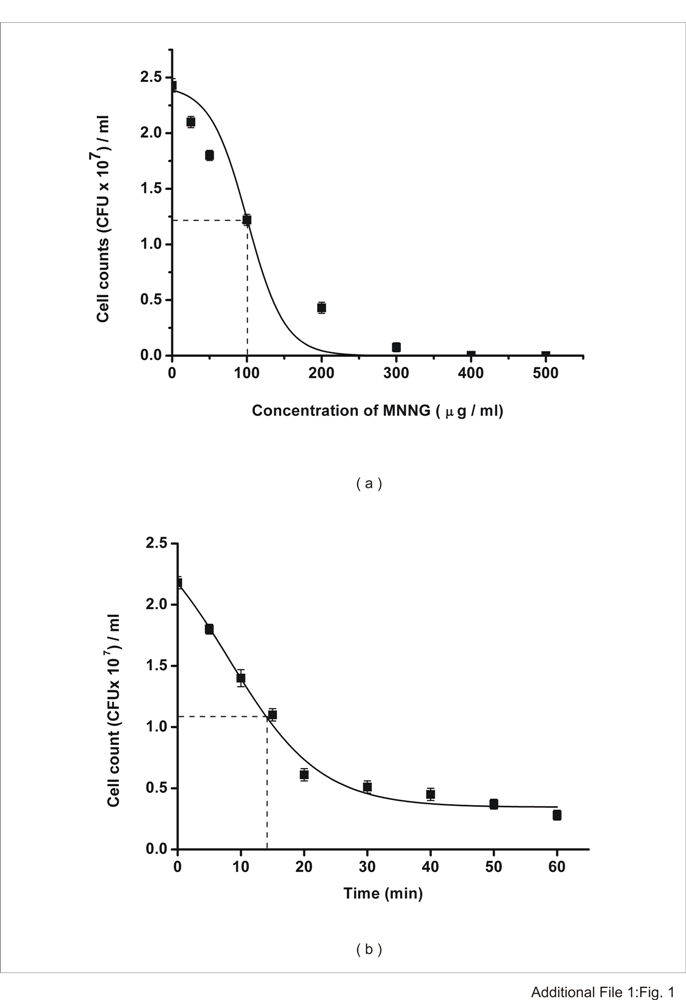

Supplement: Supplementary file 1 — Additional file 1: Figure S1. The survival curve of Y. lipolytica NCIM 3589 in presence of MNNG. (a) different concentration of MNNG (0 - 500 µg/ml). (b) different exposure time to MNNG (0-60 min). (The dotted line indicates the LD 50 value of 100 µg/ml and ~15 min). [file 12934_2017_790_MOESM1_ESM.tif]

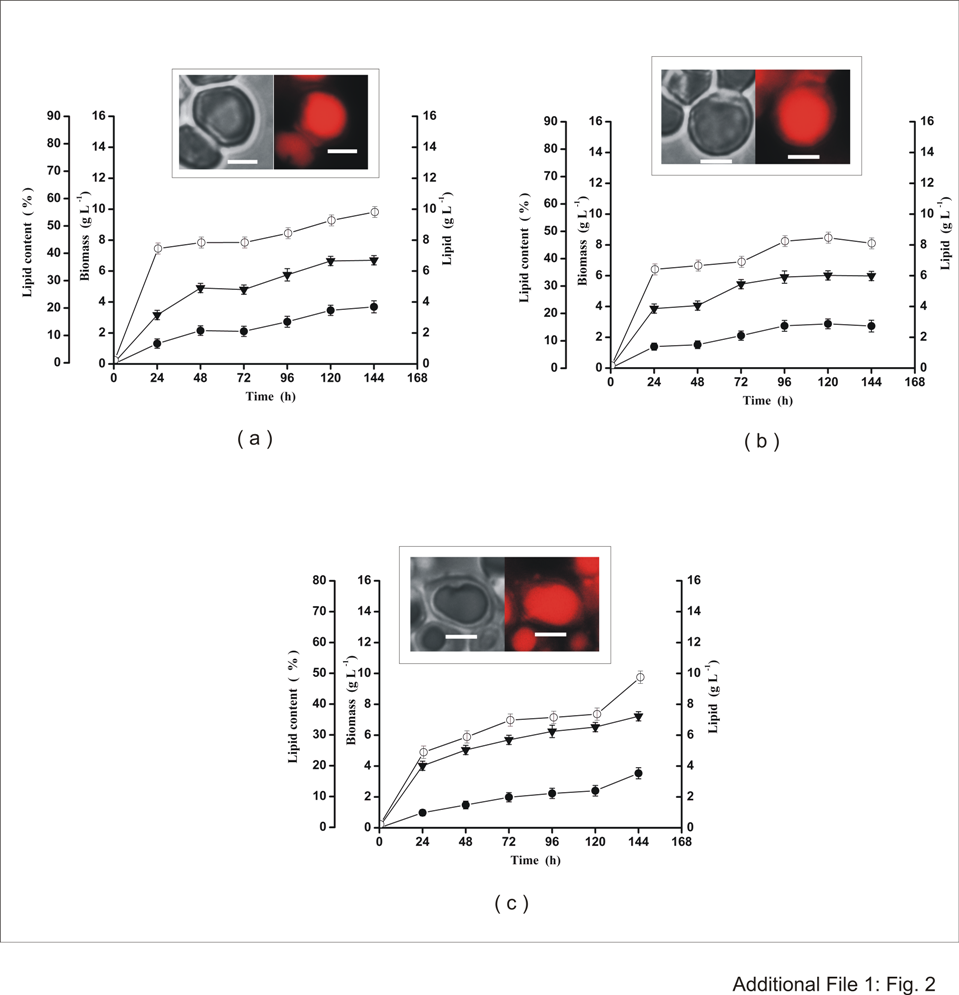

Supplement: Supplementary file 2 — Additional file 2: Figure S2.Time course studies to determine biomass, total lipid yield and lipid content of MNNG treated mutants. Mutants (a) YlC4, (b) YlC5 and (c) YlC6 were grown on 100 gL−1 WCO as mentioned in Methods. All values are represented as mean ± SD, determined after three independent experiments. Biomass (gL−1) -black down pointing triangle,Total lipid yield (gL−1) -black circle, Lipid content (%)-○. Inset: In each graph, light microscopy (left panel) and Nile red fluorescence microscopy (right panel) images of the respective Y. lipolytica mutants under 100 x oil immersion objective. Bar indicates 4 μm. [file 12934_2017_790_MOESM2_ESM.tif]

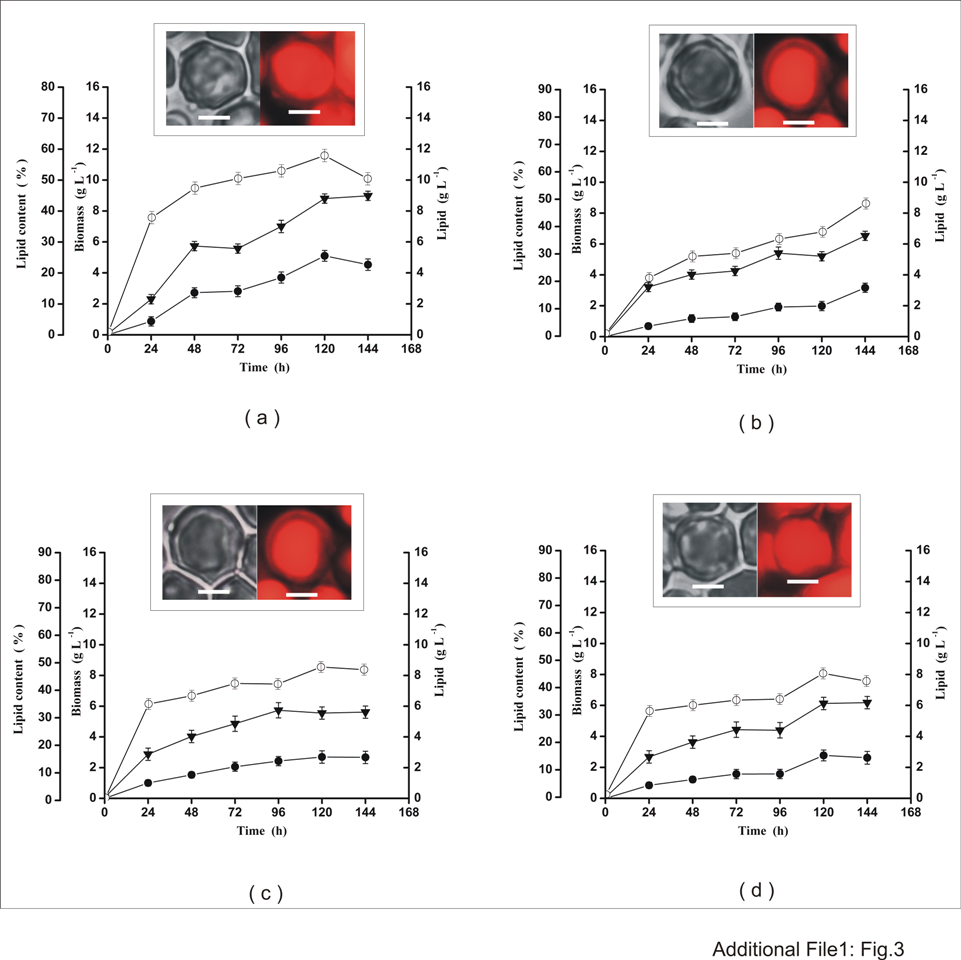

Supplement: Supplementary file 3 — Additional file 3: Figure S3. Time course studies to determine biomass, total lipid yield and lipid content of MNNG + cerulenin treated mutants. Mutants (a) YlB2, (b) YlB3, (c) YlD5 and (d) YlE2 were grown on 100 gL−1 WCO as mentioned in Methods. All values are represented as mean ± SD, determined after three independent experiments. Biomass (gL−1) -black down pointing triangle, Total lipid yield (gL−1) - black circle, Lipid content (%) - ○. Inset: In each graph light microscopy (left panel) and Nile red fluorescence microscopy (right panel) images of the respective Y. lipolytica mutants under 100 × oil immersion objective. Bar indicates 4 μm. [file 12934_2017_790_MOESM3_ESM.tif]
