# Supplementary material for: Mutants of Yarrowia lipolytica NCIM 3589 grown on waste cooking oil as a biofactory for biodiesel production
Source: Microb Cell Fact. 2017 Oct 24;16:176. doi: 10.1186/s12934-017-0790-x (PMC5655982; doi:10.1186/s12934-017-0790-x)
Supplement: Supplementary file 4 — Additional file 4: Table S1. Lipid content of the three mutants, YlB6, YlC7 and YlE1 after the 6th, 12th, 18th and 24th subculture. [file 12934_2017_790_MOESM4_ESM.docx]

**Additional File 1: Table 1 BLAST results of LSU and ITS regions for the mutants YlB6, YlC7 and YlE1**

| Mutant | Description | [Max score](http://blast.ncbi.nlm.nih.gov/Blast.cgi?CMD=Get&ALIGNMENTS=100&ALIGNMENT_VIEW=Pairwise&DATABASE_SORT=0&DESCRIPTIONS=100&DYNAMIC_FORMAT=on&FIRST_QUERY_NUM=0&FORMAT_OBJECT=Alignment&FORMAT_PAGE_TARGET=&FORMAT_TYPE=HTML&GET_SEQUENCE=yes&I_THRESH=&LINE_LENGTH=60&MASK_CHAR=2&MASK_COLOR=1&NUM_OVERVIEW=100&OLD_BLAST=false&PAGE=Nucleotides&QUERY_INDEX=0&QUERY_NUMBER=0&RESULTS_PAGE_TARGET=&RID=JGURG0V1014&SHOW_LINKOUT=yes&SHOW_OVERVIEW=yes&STEP_NUMBER=&WORD_SIZE=11&OLD_VIEW=false&DISPLAY_SORT=1&HSP_SORT=1) | [Total score](http://blast.ncbi.nlm.nih.gov/Blast.cgi?CMD=Get&ALIGNMENTS=100&ALIGNMENT_VIEW=Pairwise&DATABASE_SORT=0&DESCRIPTIONS=100&DYNAMIC_FORMAT=on&FIRST_QUERY_NUM=0&FORMAT_OBJECT=Alignment&FORMAT_PAGE_TARGET=&FORMAT_TYPE=HTML&GET_SEQUENCE=yes&I_THRESH=&LINE_LENGTH=60&MASK_CHAR=2&MASK_COLOR=1&NUM_OVERVIEW=100&OLD_BLAST=false&PAGE=Nucleotides&QUERY_INDEX=0&QUERY_NUMBER=0&RESULTS_PAGE_TARGET=&RID=JGURG0V1014&SHOW_LINKOUT=yes&SHOW_OVERVIEW=yes&STEP_NUMBER=&WORD_SIZE=11&OLD_VIEW=false&DISPLAY_SORT=2&HSP_SORT=1) | [Query cover](http://blast.ncbi.nlm.nih.gov/Blast.cgi?CMD=Get&ALIGNMENTS=100&ALIGNMENT_VIEW=Pairwise&DATABASE_SORT=0&DESCRIPTIONS=100&DYNAMIC_FORMAT=on&FIRST_QUERY_NUM=0&FORMAT_OBJECT=Alignment&FORMAT_PAGE_TARGET=&FORMAT_TYPE=HTML&GET_SEQUENCE=yes&I_THRESH=&LINE_LENGTH=60&MASK_CHAR=2&MASK_COLOR=1&NUM_OVERVIEW=100&OLD_BLAST=false&PAGE=Nucleotides&QUERY_INDEX=0&QUERY_NUMBER=0&RESULTS_PAGE_TARGET=&RID=JGURG0V1014&SHOW_LINKOUT=yes&SHOW_OVERVIEW=yes&STEP_NUMBER=&WORD_SIZE=11&OLD_VIEW=false&DISPLAY_SORT=4&HSP_SORT=0)age (%) | [E value](http://blast.ncbi.nlm.nih.gov/Blast.cgi?CMD=Get&ALIGNMENTS=100&ALIGNMENT_VIEW=Pairwise&DATABASE_SORT=0&DESCRIPTIONS=100&DYNAMIC_FORMAT=on&FIRST_QUERY_NUM=0&FORMAT_OBJECT=Alignment&FORMAT_PAGE_TARGET=&FORMAT_TYPE=HTML&GET_SEQUENCE=yes&I_THRESH=&LINE_LENGTH=60&MASK_CHAR=2&MASK_COLOR=1&NUM_OVERVIEW=100&OLD_BLAST=false&PAGE=Nucleotides&QUERY_INDEX=0&QUERY_NUMBER=0&RESULTS_PAGE_TARGET=&RID=JGURG0V1014&SHOW_LINKOUT=yes&SHOW_OVERVIEW=yes&STEP_NUMBER=&WORD_SIZE=11&OLD_VIEW=false&DISPLAY_SORT=0&HSP_SORT=0) | [Ident](http://blast.ncbi.nlm.nih.gov/Blast.cgi?CMD=Get&ALIGNMENTS=100&ALIGNMENT_VIEW=Pairwise&DATABASE_SORT=0&DESCRIPTIONS=100&DYNAMIC_FORMAT=on&FIRST_QUERY_NUM=0&FORMAT_OBJECT=Alignment&FORMAT_PAGE_TARGET=&FORMAT_TYPE=HTML&GET_SEQUENCE=yes&I_THRESH=&LINE_LENGTH=60&MASK_CHAR=2&MASK_COLOR=1&NUM_OVERVIEW=100&OLD_BLAST=false&PAGE=Nucleotides&QUERY_INDEX=0&QUERY_NUMBER=0&RESULTS_PAGE_TARGET=&RID=JGURG0V1014&SHOW_LINKOUT=yes&SHOW_OVERVIEW=yes&STEP_NUMBER=&WORD_SIZE=11&DISPLAY_SORT=3&HSP_SORT=3)ity (%) | Accession number |
| --- | --- | --- | --- | --- | --- | --- | --- |
| YlB6 LSU | *Yarrowia lipolytica* partial 26S rRNA gene, strain ZIM  2416, isolate BGGO5‐Y84 | 1150 | 1150 | 100 | 0.0 | 100 | HE660067.1 |
| YlB6 ITS | *Yarrowia lipolytica* strain UOA/HCPF 11503 isolate ISHAM-ITS_ID MITS567 18S ribosomal RNA gene, partial sequence; internal transcribed spacer 1, 5.8S ribosomal RNA gene, and internal transcribed spacer 2, complete sequence; and 26S ribosomal RNA gene, partial sequence | 621 | 621 | 100 | 3e-174 | 99 | KC254112.11 |
| YlC7  LSU | [*Yarrowia lipolytica* strain MSR 80 26S ribosomal RNA gene, partial sequence](http://blast.ncbi.nlm.nih.gov/Blast.cgi#alnHdr_160918923) | 1243 | 1243 | 100 | 0.0 | 100 | [EU256373.1](http://www.ncbi.nlm.nih.gov/nucleotide/160918923?report=genbank&log$=nucltop&blast_rank=1&RID=JH7XRV3Z014) |
| YlC7  ITS | *Yarrowia lipolytica* strain UOA/HCPF 11503 isolate ISHAM-ITS_ID MITS567 18S ribosomal RNA gene, partial sequence; internal transcribed spacer 1, 5.8S ribosomal RNA gene, and internal transcribed spacer 2, complete sequence; and 26S ribosomal RNA gene, partial sequence | 621 | 621 | 100 | 3e-174 | 99 | [KC254112.112.1](http://www.ncbi.nlm.nih.gov/nucleotide/434861228?report=genbank&log$=nucltop&blast_rank=1&RID=JGURG0V1014) |
| YlE1 LSU | [*Yarrowia lipolytica* genomic DNA containing ITS1, 5.8S rRNA gene, ITS2, 28S rRNA gene, culture collection ZIM:2498, isolate ZG7-Y193](http://blast.ncbi.nlm.nih.gov/Blast.cgi#alnHdr_410067019) | 1052 | 1052 | 100 | 0.0 | 100 | [HF545672.1](http://www.ncbi.nlm.nih.gov/nucleotide/410067019?report=genbank&log$=nucltop&blast_rank=1&RID=K640CHB1015) |
| YlE1  ITS | [*Yarrowia lipolytica* strain UOA/HCPF 11503 isolate ISHAM-ITS_ID MITS567 18S ribosomal RNA gene, partial sequence; internal transcribed spacer 1, 5.8S ribosomal RNA gene, and internal transcribed spacer 2, complete sequence; and 26S ribosomal RNA gene, partial sequence](http://blast.ncbi.nlm.nih.gov/Blast.cgi#alnHdr_434861228) | 621 | 621 | 100 | 3e-174 | 99 | [KC254112.112.1](http://www.ncbi.nlm.nih.gov/nucleotide/434861228?report=genbank&log$=nucltop&blast_rank=1&RID=JGURG0V1014) |
